# Empowering Patients and Transforming Healthcare in the Post-COVID-19 Era: The Role of Digital and Wearable Technologies

**DOI:** 10.3390/jpm13050722

**Published:** 2023-04-25

**Authors:** Shabbir Syed-Abdul, Yu-Chuan Li

**Affiliations:** 1 Graduate Institute of Biomedical Informatics, College of Medical Sciences and Technology, Taipei Medical University, Taipei 235, Taiwan; jaak88@gmail.com; 2 International Center for Health Information Technology, College of Medical Science and Technology, Taipei Medical University, Taipei 235, Taiwan

## 1. Introduction

The COVID-19 pandemic has dramatically impacted the global healthcare system, revealing critical gaps in our capacity to provide efficient and effective care to patients, particularly those with chronic diseases. As the virus continues to persist as an endemic one, it has become clear that innovative solutions are urgently needed to mitigate the long-term health consequences that come with it [1,2]. In this Special Issue of the *Journal of Personalized Medicine*, we explore the potential of digital and wearable technologies to transform healthcare delivery, empower patients, and improve outcomes in the post-COVID-19 era.

## 2. The Power of Remote Healthcare

With the ongoing threat of COVID-19, remote healthcare has emerged as a vital tool to enable continuous telemonitoring and avoid unnecessary hospital visits. The integration of digital health tools and wearable devices, such as smartwatches and fitness trackers, has allowed healthcare providers to monitor patients’ vital signs, track medication adherence, and detect early warning signs of potential health complications [3]. As a result, patients can access timely, personalized care while reducing the risk of virus transmission.

## 3. The Role of Artificial Intelligence and Machine Learning

Recent advancements in artificial intelligence (AI) and machine learning have presented new opportunities to enhance the capabilities of digital health tools and wearable technologies. By leveraging complex algorithms and large datasets, researchers are developing innovative solutions for early diagnosis, intervention, and improved healthcare delivery [2,4]. In this Special Issue, we invite submissions exploring the integration of signal processing, AI, and machine learning with mobile and wearable technology to create powerful tools for remote healthcare.

## 4. Bioinformatics and Personalized Medicine

The field of bioinformatics has the potential to revolutionize personalized medicine by providing valuable insights into patients’ unique genetic, metabolic, and physiological profiles. By combining this information with data gathered from wearable devices, healthcare providers can develop highly targeted treatment plans tailored to each individual’s specific needs [2]. We are particularly interested in research that examines the integration of bioinformatics with digital health tools to improve patient care and outcomes in the context of COVID-19.

## 5. Patient Empowerment and Engagement

A key factor in improving health outcomes for patients with chronic diseases is their ability to actively engage in their own care. Digital health tools and wearable technologies can empower patients to take charge of their own health by providing real-time feedback on their physical activity, sleep patterns, and other lifestyle factors. This information can motivate patients to adopt healthier behaviors and facilitate better communication with their healthcare providers [5]. In this Special Issue, we encourage submissions that explore the role of digital and wearable technologies in promoting patient empowerment and engagement in the post-COVID-19 era.

As digital and wearable technologies continue to evolve and play an increasingly important role in healthcare, a number of challenges and opportunities arise. In this section, we will discuss some of the most pressing issues and the potential solutions that can lead to more efficient and personalized healthcare delivery.

## 6. Implementation Challenges

Data privacy and security: The massive amount of sensitive health data collected by digital health tools and wearable devices raises concerns about data privacy and security. Ensuring that patients’ personal information is protected from unauthorized access is crucial to maintaining trust in these technologies [2].

Interoperability: The integration of various digital health tools and wearable devices with existing healthcare systems can be challenging due to differences between data formats, communication protocols, and system architectures. Interoperability is essential for seamless data exchange and efficient collaboration among healthcare providers [2].

Accessibility and the digital divide: The benefits of digital health tools and wearable technologies can only be fully realized if they are accessible to all patients, regardless of socioeconomic status or geographic location. Addressing the digital divide is critical to ensuring that these technologies do not exacerbate existing health disparities [3,6].

Regulatory and reimbursement issues: The rapid development of digital health tools and wearable technologies has outpaced that of existing regulatory frameworks, leading to uncertainty regarding the approval, oversight, and reimbursement of these innovations. Establishing clear guidelines and standards is essential for fostering the adoption of these technologies in mainstream healthcare [7].

## 7. Potential advantages and Opportunities

Personalized healthcare delivery: Digital health tools and wearable devices offer the opportunity to tailor healthcare interventions to each individual’s unique needs, leading to more effective treatment strategies and improved patient outcomes [2].

Enhanced patient engagement: By providing patients with real-time feedback on their health status, digital health tools and wearable devices can foster increased patient engagement and motivate individuals to adopt healthier behaviors [5].

Improved healthcare efficiency: The integration of digital health tools and wearable technologies can streamline healthcare processes, reduce administrative burdens, and minimize the risk of medical errors, ultimately leading to more efficient and cost-effective care [6].

Early detection and intervention: The continuous monitoring capabilities of digital health tools and wearable devices can enable healthcare providers to identify potential health issues at an early stage, facilitating the implementation of timely interventions and potentially preventing the progression of chronic diseases [3].

Remote monitoring and telehealth: In the post-COVID-19 era, digital health tools and wearable devices can facilitate remote monitoring and telehealth services, reducing the need for in-person visits and minimizing the risk of virus transmission [3,7].

## 8. Summary

As the world continues to combat the enduring effects of COVID-19, there is a pressing need for innovative solutions to be developed to help patients with chronic diseases survive alongside the virus. The rapid advancement in information and communication technologies presents a unique opportunity to transform healthcare delivery, improve patient outcomes, and empower individuals to take control of their health. In this Special Issue, we welcome original articles, reviews, perspectives, and communications that elucidate the role of digital and wearable technologies in the COVID-19 pandemic and beyond.

By addressing these challenges and capitalizing on the opportunities presented by digital and wearable technologies, we can pave the way for a more efficient, personalized, and patient-centered healthcare system in the post-COVID-19 era. This Special Issue aims to explore innovative solutions and strategies to overcome these challenges and harness the potential of these technologies to transform healthcare for the better.
